# Effects of high hydrostatic pressure on the rheological properties and foams/emulsions stability of *Alyssum homolocarpum* seed gum

**DOI:** 10.1002/fsn3.1834

**Published:** 2020-09-01

**Authors:** Sajad Ghaderi, Mohammad Ali Hesarinejad, Elhamalsadat Shekarforoush, Seyyed Mahdi Mirzababaee, Farzad Karimpour

**Affiliations:** ^1^ Faculty of Health and Nutrition Yasuj University of Medical Sciences Yasuj Iran; ^2^ Department of Food Processing Research Institute of Food Science & Technology (RIFST) Mashhad Iran; ^3^ Department of Food Science University of Copenhagen Copenhagen Denmark; ^4^ Department of Food Machinery Design Research Institute of Food Science & Technology (RIFST) Mashhad Iran; ^5^ Social Determinants of Health Research Center Yasuj University of Medical Sciences Yasuj Iran

**Keywords:** *Alyssum homolocarpum* seed gum, functional properties, high hydrostatic pressure, hydrocolloid, rheology

## Abstract

The ability to modify food and increase the shelf life by enhanced stability using nonthermal process is of interest to many food companies. Here, we investigate the effects of high hydrostatic pressure (HHP), as a nonthermal process, at various pressure levels (200, 400, and 600 MPa for 30 min) on the functional properties of aqueous dispersions of *Alyssum homolocarpum* seed gum (AHSG). In this regard, the rheological properties, foam stability, and emulsion stability of the HHP‐treated gums were analyzed and compared. Dynamic oscillatory test indicated that the HHP‐treated gums had more gel‐like behavior than viscous‐like behavior (storage modulus > loss modulus) at designated pressures. When AHSG was treated by HHP, both elastic (G′) and viscous (G″) moduli were increased compared to the native AHSG. The native‐ and HHP‐treated gums exhibited a shear‐thinning (pseudoplastic) behavior. Furthermore, the pressure levels have a significant effect on consistency coefficient, flow behavior index, and yield stress (*p* < .05) of AHSG. The results showed that the HHP‐treated gums lead to improve the foam and emulsion stability of AHSG. Finally, we assume that HHP‐treated AHSG improves texture in the food materials.

## Graphical abstract

When AHSG was treated by HHP, both elastic and viscous moduli were increased compared the native AHSG. The pressure levels have a significant effect on consistency coefficient, flow behaviour index, and yield stress (*p* < .05) of AHSG. The results showed that the HHP‐treated gums lead to improve the foam and emulsion stability of AHSG.

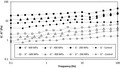

## 1 INTRODUCTION

High pressure processing is also called high hydrostatic pressure (HHP), ultra‐high pressure processing, pascalization, or cold pasteurization (Daher, Le Gourrierec, & Pérez‐Lamela, 2017). It has been widely explored and has indicated a major potential for various type of food processing applications in recent years (Vatankhah, Taherian, & Ramaswamy, 2018). It is a food processing technology which employs high pressure to solid or liquid foods to improve their safety and, in some cases, organoleptic properties and quality (Daher et al., 2017). HHP has been successfully proven to be used as an alternative to conventional heat treatments (Franchi, Tribst, & Cristianini, 2013; Pinho, Oliveira, Leite Júnior, Tribst, & Cristianini, 2015) or in combination with mild temperatures (Ferragut et al., 2015) in some beverages. HHP is also an emerging process to modify food biopolymers relies on the achievement and control of pressure‐induced changes in the molecular structure (Olsen & Orlien, 2016). This includes starch gelatinization and protein denaturation or aggregation to improve the texture of foods (Knorr, Heinz, and Buckow (2006); Messens, Van Camp, & Huyghebaert, 1997; Molina, Papadopoulou, & Ledward, 2001; Li et al., 2011). High pressure is a rather useful tool by which the texture of foodstuff can be manipulated (Ledward, 1995). Several studies have represented the impacts of HHP on the rheological and functional properties of macromolecules associated with various food matrices (Ahmed, Ramaswamy, Ayad, Alli, & Alvarez, 2007; Laneuville, Turgeon, & Paquin, 2013; Panteloglou, Bell, & Ma, 2010; Vatankhah et al., 2018; Xue et al., 2018).

Seed gums are polysaccharides from the vegetal origin and are widely employed in the food and chemical industries as thickeners, stabilizers, gelling agents, and emulsifiers (Alizadeh Behbahani et al., 2017; Belmiro, Tribst, & Cristianini, 2018; Hesarinejad, Koocheki, & Razavi, 2014a). Regarding the structure and texture of food products, the rheological and functional properties of hydrocolloids are of utmost importance (Hesarinejad, Razavi, & Koocheki, 2015a). The choice of a specific gum depends on its application and purpose because each form of gum has particular values with respect to viscosity, intrinsic viscosity, stability, and emulsifying and gelling properties, with these parameters being determined by its structure (Belmiro et al., 2018). HHP is able to alter those properties positively by inducing changes in the original polymer, allowing for new applications and improvements with respect to the technical properties of gums (Belmiro et al. (2018). The effect of HHP on food hydrocolloids depends on the pressure amplitude, hydrocolloid type and concentration, pressurization time, temperature, and media (Pei‐Ling, Xiao‐Song, & Qun, 2010; Porretta, Birzi, Ghizzoni, & Vicini, 1995).

*Alyssum homolocarpum* is natives of some Middle Eastern countries like Pakistan, Iran, Iraq, Saudi Arabia, and Egypt (Amin, 2005). *Alyssum homolocarpum* seed gum (AHSG) has been used as medicinal remedies. AHSG exhibited non‐Newtonian, pseudoplastic behavior over a range of 1.5%–4% at 5°C–65°C (Koocheki, Mortazavi, Shahidi, Razavi, & Taherian, 2009). The elastic component of AHSG has always been higher than the viscous one at concentrations of 1.5%–3%, which means that AHSG has had weak gel‐like properties (Hesarinejad, Koocheki, & Razavi, 2014b). AHSG is highly purified and contains 85.33% carbohydrate with a small amount of uronic acid (5.63%) (Hesarinejad, Razavi, & Koocheki, 2015b). AHSG has a low molecular weight (3.66 × 10^5^ Da) with relatively flexible chain and medium intrinsic viscosity (18.34 dl/g) at ambient temperature (Hesarinejad et al., 2015b). The electrostatic interaction and particle size of AHSG solution were − 25.81 mV (at neutral pH) and 225.36 nm, respectively (Hesarinejad et al., 2015b). The major monosaccharide compositions of AHSG are galactose (82.97%), glucose (5.7%), rhamnose (5.04%), xylose (2.72%), mannose (3.04%), and arabinose (0.53%), and it is likely a galactan‐type polysaccharide (Hesarinejad et al., 2015b). AHSG behaves like a typical polyelectrolyte because of the presence of carboxyl and hydroxyl groups (Hesarinejad et al., 2015b). AHSG can be applied for thickening, suspending, stabilizing, and as a gelling agent (Koocheki & Hesarinejad, 2019).

The rheological properties of food hydrocolloids are forcefully influenced by temperature, pressure, concentration, and physical state of dispersion (Van Vliet & Walstra, 1980). Owing to the difference in extrinsic conditions within the fluid food systems, the benefits of AHSG solutions will be changed from one situation to another. Therefore, the aim of this work was to study the simultaneous effect of high hydrostatic pressure level (200–600 MPa) on the rheological and functional properties of this gum.

## 2 MATERIALS AND METHODS

### 2.1 Materials

The AHSG was extracted and purified according to Koocheki et al. (2010). AHSG dispersion at 1% (w/w) was prepared by adding an appropriate amount of freeze‐dried AHSG powder to a portion of deionized water that contained 0.02% sodium azide as an antimicrobial preservative. Then, these dispersions stirred for 2 hr on a magnetic stirrer at ambient temperature and put on roller shaker overnight to ensure complete hydration. This sample was kept at refrigerator before carrying out the experiments. All chemicals used in this study were of analytical grade and purchased from Merck (Darmstadt, Germany) company.

### 2.2 High hydrostatic pressure treatment

**Figure 1:**
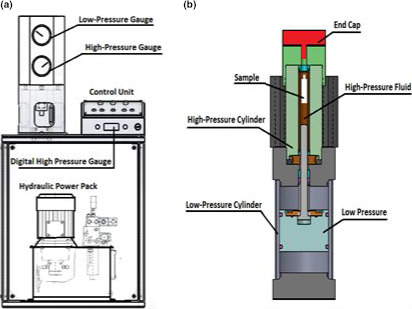
Schematic of the high pressure system (a); and schematic of the sample holder and its position in the high pressure machine (b)

A hydrostatic pressurization unit (RIFST, Mashhad, Iran) with a chamber volume of 120 ml was applied to generate high pressure levels (Figure 1). The device has the ability to adjust the pressure level through a manual valve control. The pressure level is visible both in analogue and in digital, and the permissible pressure is controlled through a pressure transducer and a pressure relief valve. The pressure applied to the samples was 200, 400, and 600 MPa, and a pressure rise of 10 MPa.s^‐1^ was implemented, and also, the decompression time was less than 5 s. The time is taken to apply the samples 30 min. The samples were stored at a temperature of 4°C before loading in the refrigerator and did not have a significant change in pressure applied to the samples (the maximum measured temperature was 23°C for a sample of 600 MPa). Triplicate samples were applied for each treatment. Untreated AHSG was used as control.

### 2.3 Measurement of rheological properties

Steady and dynamic shear measurements were conducted with a Physica MCR301 controlled stress/strain rheometer (AnTon paar GmbH, Germany) using a parallel plate geometry (50 mm diameter). After transferring the sample onto the rheometer plate, the minimum gap was adjusted to 1.0 mm. The excess material was wiped off with a spatula, and the edges were coated with a thin layer of silicone oil to reduce evaporation during measurements. After loading, the sample was allowed to relax for 1 min before the measurements. The linear viscoelastic region (LVR) of pressurized‐AHSG was determined by performing an amplitude sweep tests. Frequency sweep measurements at a very low strain of 0.1% (LVR) were carried out where it approaches linear behavior. The mechanical spectra were characterized by values of G′ and G″ as a function of frequency in the range of 0.1–10 Hz at 25°C. Steady flow behavior of pressurized‐AHSG dispersions was measured over a range of shear rates from 0.1 to 300 s^‐1^ with a linearly increasing scale. The apparent viscosity at a given shear rate was determined as the ratio of shear stress to shear rate (Steffe, 1996). The Rheoplus/32 software V3.40 was employed for data evaluation. At least, triplicate of each measurement was made.

In order to perform a quantitative comparison of pressurized‐AHSG dispersions, six rheological flow models based on shear stress–shear rate were measured (Ostwald–Waele, Bingham, Herschel–Bulkley, Casson, National Confectioners Association/CMA Casson and Vocadlo). The best fit model was selected according to the determination coefficient (R^2^) and root mean square error (RMSE).

Ostwald–Waele's (or Power law) model:

(1) $$\tau =k{\dot{\gamma }}^{n}$$

where k is the consistency coefficient (Pa.s^n^), and n is the flow behavior index for Ostwald–Waele model.

Bingham's model:

(2) $$\tau =\tau_{0}+\eta \dot{\gamma }$$

where η is called the Bingham plastic viscosity (Pa.s) and τ_0_ is the yield stress (Pa).

Herschel–Bulkley's model:

(3) $$\tau =\tau_{0}+k{\left(\dot{\gamma }\right)}^{n}$$

where τ_0_ is the yield stress (Pa), k is the consistency coefficient (Pa.s^n^), and n is flow behavior index for Herschel–Bulkley model.

Casson's model:

(4) $$\tau^{0.5}=\tau_{0}^{0.5}+k{\left(\dot{\gamma }\right)}^{0.5}$$

where $\tau_{0}^{0.5}$ (Pa^0.5^) and k (Pa^0.5^s^0.5^) are the intercept and slope of plot of τ^0.5^ versus ${\dot{\gamma }}^{0.5}$, respectively. Then, the magnitudes of τ_0_ and k have been used as the Casson yield stress (Pa) and Casson plastic viscosity (Pa.s), respectively.

NCA/CMA Casson's model:

(5) $$\surd \tau =\surd \tau_{0}+\surd \eta \surd \dot{\gamma }$$

where τ_0_ is the Casson yield value (Pa), and η is the Casson plastic viscosity (Pa.s).

Vocadlo's model:

(6) $$\tau ={\left(\tau_{0}^{\frac{1}{n}}+k\dot{\gamma }\right)}^{n}$$

where τ_0_ is the yield stress (Pa), k is the consistency coefficient (Pa^1/n^s^n^), and n is the flow behavior index.

### 2.4 Emulsion preparation and characterization

The oil in water emulsions (30:70 v/v) were prepared by adding 18 ml of sunflower oil into 60 ml of pressurized‐AHSG dispersions (1%w/v) while mixing by a mechanical high speed stirrer (2000 rpm). After mixing (3 min), the suspension was homogenized by Ultra‐Turrax T‐25 homogenizer (IKA Instruments, Germany) at 20,000 rpm for 6 min in alternate cycles of homogenization for 1 min and rest for 2 min at ambient temperature. Prepared emulsions were then centrifuged at 2000× g for 10 min. Emulsion stability was calculated based on the following equation (Sciarini, Maldonado, Ribotta, Pérez, & León, 2009):

(7) $$\text{Emulsion}\;\text{Stability}=\frac{f_{\mathrm{ev}}}{i_{\mathrm{ev}}}\times 100$$

where *f_ev_* is the final emulsion volume and *i_ev_* is initial emulsion volume.

The size distribution of the pressurized‐AHSG emulsions at various pressure level (200–600 MPa) was determined by a laser diffraction particle sizer (Fritsch Particle sizer Analysette 22, Fritsch Co., Germany) at 25°C. The refractive index of the solvent was equal to 1.33. The average sizes of particles were measured by light beam scattering at a wavelength of 633 nm. Three measurements of the particle size were made, and the average value is reported.

### 2.5 Foaming and foam characterization

0.3% (w/v) ovalbumin (Applichem, USA) was added to the 20 ml of diluted pressurized‐AHSG dispersions (0.1%w/v) while whipping strenuously at 15,000 rpm for 2 min with a homogenizer (Ultra‐Turrax T‐25, Heidolph, Germany) (Koocheki, Razavi, & Hesarinejad, 2012). The foam stability was calculated using the following equation:

(8) $$\text{Foam}\;\;\text{Stability}\left(\%\right)=\frac{f_{\mathrm{fv}}}{t_{\mathrm{sv}}}\times 100$$

where *f_fv_* is the foam volume after 30 min and *t_sv_* is total suspension volume.

### 2.6 Statistical analyses

Statistical analysis was performed using SPSS software (SPSS 24.0 for Windows, SPSS Inc., Chicago, IL, USA). Significant difference at 95% confidence level was evaluated using Duncan's multiple range test to compare the treatment means.

## 3 RESULTS AND DISCUSSIONS

### 3.1 Effects of HHP on rheological properties of AHSG

Applied pressure plays the main role in the rheological and textural characteristics in foods. HHP has been modified the rheological and textural properties of food proteins and hydrocolloids (Ahmed & Ramaswamy, 2004; Ahmed, Ramaswamy, Alli, & Ngadi, 2003; Ahmed et al., 2007; Alvarez, Ramaswamy, & Ismail, 2008; Belmiro et al., 2018). The rheological parameters of pressurized‐AHSG dispersions at the different hydrostatic pressure levels obtained by fitting the shear stress–shear rate data to the various time‐independent rheological models are shown in Table 2. Based on the Herschel–Bulkley and Vocadlo models, pressurized‐AHSG dispersions demonstrated a non‐Newtonian shear‐thinning fluid with the presence of yield stress at all hydrostatic pressure tested. Among rheological models, the Herschel–Bulkley model founded best, with higher R^2^ and lower RMSE. This was in agreement with results of Koocheki & Razavi (2009), who reported that Herschel–Bulkley equation well described the flow behavior of AHSG dispersion (Koocheki & Razavi, 2009). Similar this was also reported for pressurized‐Xanthan gum by Ahmed & Ramaswamy (Ahmed & Ramaswamy, 2004). On the contrary, Koocheki et al. (2009) and Anvari et al. (2016) showed that the flow behavior was described with Ostwald power law and Carreau models, respectively (Anvari et al., 2016; Koocheki et al., 2009). This difference could be due to pressure application, range of shear rate, source of seed, etc.

The *k* value has been considered as an important quality factor in food processing among the rheological parameters. Table 2 represented that pressure level had a significant effect on the consistency coefficient (*k*). The pressure affected the *k* value of AHSG (Table 2). The result demonstrated that the magnitude of *k* increased with an increase in pressure level. As pressure level elevated, the AHSG chains probably come closer and resulting in their mutual entanglement. This increment of consistency coefficient was also possibly due to the decreasing particle size of gum. The highest consistency coefficient for pressurized‐AHSG dispersion was shown when the pressure treated was the highest level (600 MPa).

The values of flow behavior indices (*n*) ranged between 0.20 and 0.43 and displayed shear‐thinning nature of pressurized‐AHSG dispersions at all measurement conditions. The lower *n* values represented a greater departure from Newtonian behavior (Koocheki et al., 2010). To prepare high viscosity and good mouthfeel, hydrocolloids which have a low *n* value are desirable (Marcotte, Hoshahili, & Ramaswamy, 2001).

Increase in pressure level from 200 to 600 MPa slightly decreased the *n* value resulted in an augmentation of pseudoplasticity (Table 2). As lower values of *n* represent a pseudoplastic behavior of gum, it can be concluded that HHP treatment tends to induce higher pseudoplasticity. It has been also reported that the value of *n* and its changes is strongly dependent on molecular size (Hamza‐Chaffai, 1990). Among all samples, HHP‐treated AHSG dispersions at the maximum pressure level (600 MPa) had the highest consistency coefficient and shear‐thinning behavior (Table 1).

**Table 1:** Shear rate dependency of AHSG dispersions at different pressures using various rheological flow functions

Models	Pressure levels (MPa)			
	0.101	200	400	600
Ostwald–Waele				
*k*	3.03	2.99	3.21	4.28
*n*	0.26	0.23	0.25	0.20
R^2^	0.98	0.98	0.99	0.91
RMSE	0.233	0.174	0.186	0.403
Bingham				
*η*	0.060	0.049	0.061	0.078
τ_0_	5.00	4.75	5.26	5.90
R^2^	0.94	0.88	0.92	0.66
RMSE	0.362	0.449	0.438	0.789
Herschel–Bulkley				
*k*	1.00	2.17	2.99	4.28
*n*	0.43	0.30	0.23	0.20
τ_0_	1.29	2.09	2.72	3.00
R^2^	0.99	0.99	0.99	0.98
RMSE	0.119	0.175	0.174	0.211
Vocadlo				
*k*	16.24	19.10	25.37	28.13
*n*	0.30	0.29	0.29	0.28
τ_0_	3.39	4.01	4.31	4.35
R^2^	0.98	0.98	0.99	0.97
RMSE	0.196	0.181	0.175	0.231
Casson				
*η*	0.130	0.113	0.130	0.133
τ_0_	3.72	3.63	3.93	4.68
R^2^	0.98	0.94	0.97	0.79
RMSE	0.221	0.305	0.268	0.620
NCA/CMA Casson				
*η*	0.017	0.016	0.017	0.017
τ_0_	3.72	3.85	3.93	3.98
R^2^	0.98	0.96	0.97	0.95
RMSE	0.221	0.213	0.268	0.210

**Figure 2:**
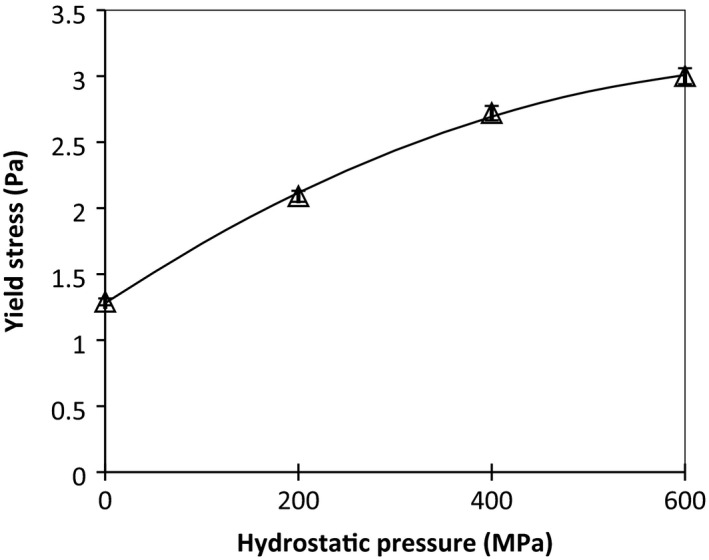
Effect of high pressure on yield stress of AHSG at different pressures

Yield stress, which results from the formation of a weak network in the gum solution, is one of the momentous qualitative factors for determining the properties of hydrocolloids (Hesarinejad et al., 2015a). The value of yield stress represented the finite stress required of hydrocolloids to initiate flow (Ahmed & Ramaswamy, 2004). It has an enormous range of practical applications in foods such as coating, spread ability, firmness of gels, and mouthfeel (Ahmed & Ramaswamy, 2004; Sun & Gunasekaran, 2009). The yield stress for native AHSG is 1.29 Pa, while this varied from 2.09 to 3 Pa as a result of the HHP treatment. The yield stress slightly increased with pressure for AHSG and followed polynomial models of order 2 (Figure 2). A similar trend was reported by Ahmed, Ramaswamy, & Hiremath (2005) for *Alphanso* pulp (Ahmed, Ramaswamy, & Hiremath, 2005). This observation could be due to the structural arrangement and intramolecular interactions of AHSG. Further investigations of intramolecular AHSG interactions at other HHP conditions may be beneficial to fully explore the impact of these factors on the rheological behavior (Ahmed et al., 2005).

**Figure 3:**
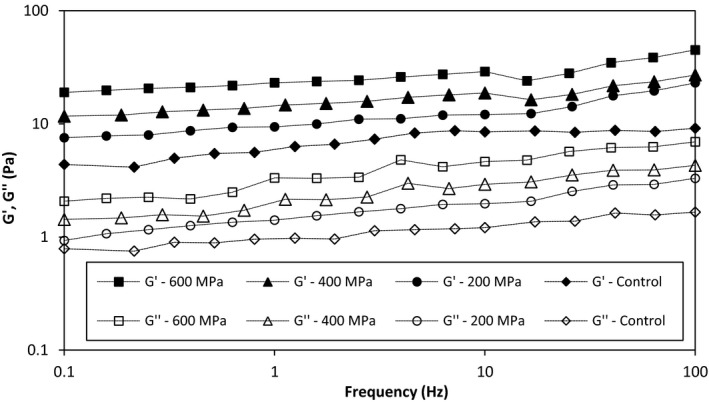
Effect of high hydrostatic pressure levels on storage modulus (closed symbols) and loss modulus (open symbols) of AHSG dispersions as functions of frequency

The frequency sweep test is used to characterize and classify the dispersions (Hesarinejad et al., 2018). Figure 3 indicates the changes in storage modulus (G′) and loss modulus (G″) as a function of frequency (Hz) and high hydrostatic pressure (MPa). They were measured over the frequency range of 0.1–10 Hz at a strain level of 0.1%. This strain was found to be within the linear viscoelastic region of the pressurized and unpressurized‐AHSG samples tested. In dilute solutions, the storage modulus is less than the loss modulus and approaches each other at higher frequencies, while in the gel‐like systems, the elastic modulus is higher than the loss modulus at all of the studied frequency domain. In addition, in concentrated polymer solutions, at low frequencies, the elastic modulus is less than the viscous modulus and intersects the middle of the frequency (Hesarinejad, Shekarforoush, Attar, & Ghaderi, 2019). As previously reported, AHSG solution at high concentration (>0.7%) has a typical weak gel‐like behavior where the magnitudes of G′ and G′′ slightly elevated with increasing frequency, having small frequency dependence (Alaeddini, Koocheki, Mohammadzadeh Milani, Razavi, & Ghanbarzadeh, 2017; Hesarinejad et al., 2015a). At low frequencies, G' values were much greater than G''. With increasing frequency, the magnitude of loss modulus increased rapidly and became closer to storage modulus. This phenomenon also displayed weak gel behavior for the AHSG samples. Similar this has been reported by Karazhiyan et al. (2011) and Hesarinejad et al. (2014) for *Lepidium sativum* and *Lepidium perfoliatum* seed gums, respectively (Hesarinejad et al., 2014a; Karazhiyan et al., 2011).

The effect of high hydrostatic pressure treatment on the viscoelastic behavior of AHSG is shown in Figure 3. At the range of pressure studied (200–600 MPa), the viscoelastic moduli of pressurized‐AHSG were higher than those of nonpressurized‐AHSG, depicting that AHSG had pressure sensitivity, and thus, it is attended that this gum can be used as a new source of a thickening agent in the food and pharmaceutical formulation, which requires the enhancement of viscoelastic properties after application high pressure. This observation can also be related to the ever‐increasing complex structure at higher pressures.

**Figure 4:**
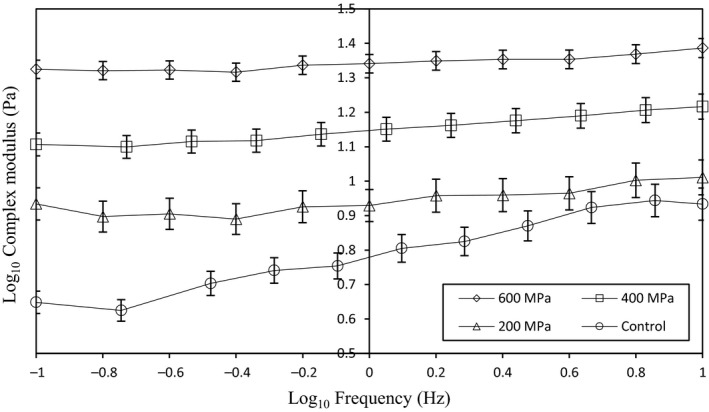
Complex modulus (G*) as a function of frequency for native and pressurized‐AHSG over the range 0.1–10 Hz at a strain of 0. 1%. Error bars extend one SE above and below the mean

A more detailed examination of the structures obtained after the pressurization of AHSG is given in Figure 4. This indicates the complex modulus (G*) as a function of frequency for both pressurized and native AHSG. High pressure treatment could also enhance the overall dynamic moduli (G*) of AHSG. This would suggest that pressure treatment may enhance overall consistency (similar values of G*) of the AHSG. These changes should be caused by an underlying structure/interaction after pressure treatment (Panteloglou et al., 2010). A similar was reported by Panteloglou et al., (2010) for pressurized hydrated gum Arabic samples (Panteloglou et al., 2010).

### 3.2 Effect of HHP on emulsion characterization

Evaluation of the particle size distribution of emulsion is very momentous as it influences the viscosity of this system (Venugopal & Abhilash, 2010). The particle size distribution of the pressurized‐AHSG emulsions was set using a Laser Particle Size Analyzer (Fritsch Analysette 22—Nanotec, Idar‐Oberstein, Germany). These data are tabulated in Table 2. In this study, d_4,3_ represents the volume mean diameter and d_3,2_ represents the area mean diameter. Table 2 exhibits a decrease in d_4,3_ and d_3,2_ with high hydrostatic pressure treatment (*p* < .05). For the pressurized‐AHSG emulsion, volume mean diameter decreased progressively with increasing pressure so that the size was reduced to about 158.4 nm (~62% of the initial size) after treatment at 600 MPa. The area mean diameter of native AHSG was approximately 223.3 nm. Following HHP treatment at 600 MPa, the area mean diameter was 125.1 nm, indicating the increased number of small particles. These results were in agreement with the results reported by Anema (2008), who reported the particle size of casein was decreased after 600 MPa of pressure treatment (Anema, 2008). Smaller particle size can lead to a more stable emulsion (Samavati, Razavi, & Mousavi, 2008), showing the pressurized‐AHSG emulsions might be more stable than the native AHSG.

The centrifugation assay is a rapid procedure for the evaluation of emulsion stability. Based on Stokes' law, the stability of the emulsion to gravitational separation can be increased by increasing the viscosity of the aqueous continuous phase (McClements, 2015). Hydrocolloids could improve the viscosity of aqueous continuous phase and precipitate or absorb onto oil droplets. The food gums could form a solid‐like structure and thicker stabilizing layer and protects oil droplets against flocculation and coalescence by modifying the rheological behavior of the aqueous phase between dispersed particles or droplets (Dickinson, 2003; Huang, Kakuda, & Cui, 2001; Imeson, 2000). Emulsion stability of pressurized‐AHSG as a function of high pressure level was shown in Table 1. The result was shown that the emulsion contains pressurized‐AHSG can be stable at all tested conditions during centrifugation. As the pressure level increased, the particle size and viscosity of the AHSG dispersions decreased and increased, respectively. Therefore, enhancing the pressure level increases the emulsion stability of AHSG. According to our observations, the size of gum particle had a direct effect on viscosity and lower particle size of AHSG improved viscosity of continuous phase and imparted more emulsion stability. The highest increase in emulsion stability was observed for emulsions containing 1% AHSG treated at 600 MPa, which increased emulsion stability by 27% compared to the control sample (Table 2). This result demonstrated that rheological characteristics are not the reason for the stability of AHSG emulsion merely. Therefore, the stability of an emulsion can also be affected by the size of the gum particle.

**Table 2:** Diameters and emulsion stability of AHSG emulsions as a function of high hydrostatic pressure levels

Pressure (MPa)	Diameters (nm)		Emulsion stability (%)
	d_3,2_	d_4,3_	
0.101	223.3 ± 0.2^a^	420.8 ± 0.8^a^	93.1 ± 1.0^c^
200	217.8 ± 0.5^a^	254.0 ± 0.6^b^	95.9 ± 0.9^b^
400	188.4 ± 0.3^b^	172.3 ± 0.6^c^	97.1 ± 1.1^b^
600	125.1 ± 0.3^c^	158.4 ± 0.4^d^	100 ± 0.7^a^

Note

Different letters within columns present significant differences (*p* < .05).

### 3.3 Effect of HHP on foam stabilization of AHSG

**Figure 5:**
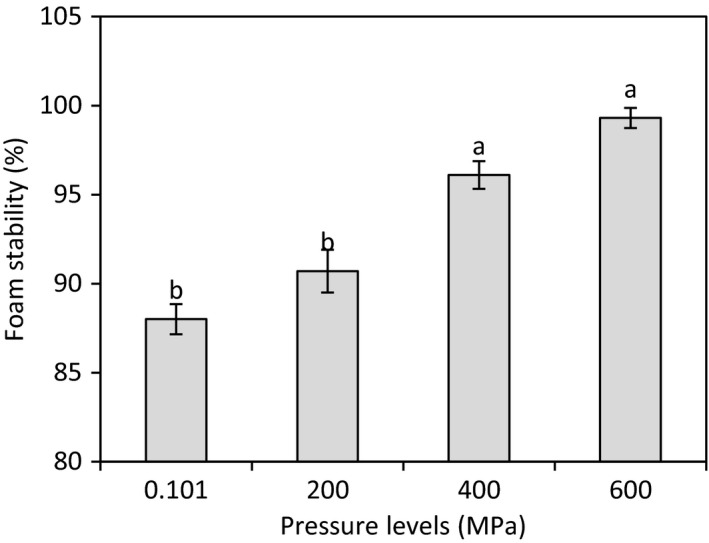
Foam stability of AHSG dispersions as a function of high hydrostatic pressure levels

Foam colloidal systems contain gas bubbles that are randomly dispersed in an aqueous phase. Instability of foams arises when the bubbles tend to join and create large bubbles. This coalescence phenomenon is accelerated by drainage of water between the bubbles (Kalsbeek & Prins, 1999). Hydrocolloids could improve foam stability by thickening or gelling roles (Dickinson, 2010). These impart positive effects on foaming properties in foods because of their high viscosity (Mott, Hettiarachchy, & Qi, 1999; Xie & Hettiarachchy, 1998). As shown in Figure 5, the effect of the hydrostatic pressure level was significant (*p* < .05) on the foam stability of pressurized‐AHSG. As hydrostatic pressure increased up to 600 MPa, foam stability of pressurized‐AHSG increased gradually. The highest foam stability of pressurized‐AHSG was obtained at the maximum pressure level of 600 MPa which had the highest consistency coefficient. The viscosity of pressurized‐AHSG dispersions was highly correlated with foam stability (r = 0.96, *p* < .001) (data not shown). The foam stability of pressurized‐AHSG at the hydrostatic pressure of 600 MPa was 40% better than that of 0.101 MPa. Hence, pressurized‐AHSG has the potential to be applied in foods to modify foaming properties due to its appropriate foaming stability and relatively high viscosity.

## 4 CONCLUSION

The high pressure processing, as an economical and environmentally friendly technology, has many applications in the food industry (preservation, color and nutritional maintenance, functional properties modifications) (Tan, 1990). In this study, it was experimentally proved that it is possible to apply HHP to change the rheological and functional properties of AHSG. The particle size of AHSG emulsion decreased steadily with increasing pressure. The maximum particle size reduction was observed at a pressure of 600 MPa. The results showed also that the emulsion stability was challenged after HHP treatment since particle size plays a predominant role in deciding the emulsion stability. The results represented that the pressurized‐AHSG showed more emulsion and foam stability. The storage and loss moduli of pressurized‐AHSG were higher than those of nonpressurized‐AHSG. The pressurized‐AHSG showed shear‐thinning behavior with yield stress which described by Herschel–Bulkley model. It was indicated that pressure level and concentration have a profound effect on *n*, *k,* and yield stress of AHSG. The HHP intensified the pseudoplasticity of AHSG which causes to make a supreme mouthfeel in the foods contain it. Regarding the change of the rheological and functional characteristics of AHSG, HHP treatment at 600 MPa for 30 min would be recommended to improve these properties. The promising findings of this work should encourage further research on using HHP or combining with other emerging technologies to improve the rheological and functional properties of hydrocolloids.
